# 28-country global study on associations between cultural characteristics and Recovery College fidelity

**DOI:** 10.1038/s44184-024-00092-9

**Published:** 2024-10-08

**Authors:** Yasuhiro Kotera, Amy Ronaldson, Daniel Hayes, Holly Hunter-Brown, Merly McPhilbin, Danielle Dunnett, Tesnime Jebara, Simran Takhi, Takahiko Masuda, Elizabeth Camacho, Ioannis Bakolis, Julie Repper, Sara Meddings, Vicky Stergiopoulos, Lisa Brophy, Clara De Ruysscher, Michail Okoliyski, Petra Kubinová, Lene Eplov, Charlotte Toernes, Dagmar Narusson, Aurélie Tinland, Bernd Puschner, Ramona Hiltensperger, Fabio Lucchi, Yuki Miyamoto, Stynke Castelein, Marit Borg, Trude Gøril Klevan, Roger Tan Boon Meng, Chatdanai Sornchai, Kruawon Tiengtom, Marianne Farkas, Hannah Moreland Jones, Edith Moore, Ann Butler, Richard Mpango, Samson Tse, Zsuzsa Kondor, Michael Ryan, Gianfranco Zuaboni, Dan Elton, Jason Grant-Rowles, Rebecca McNaughton, Charlotte Hanlon, Claire Harcla, Wouter Vanderplasschen, Simone Arbour, Denise Silverstone, Ulrika Bejerholm, Candice Powell, Susana Ochoa, Mar Garcia-Franco, Jonna Tolonen, Caroline Yeo, Ashleigh Charles, Claire Henderson, Mike Slade

**Affiliations:** 1 School of Health Sciences, Institute of Mental Health, University of Nottingham, Nottingham, NG7 2TU UK; 2 Center for Infectious Disease Education and Research, Osaka University, Osaka, Suita 565-0871 Japan; 3 Health Service and Population Research Department, King’s College London, Institute of Psychiatry, Psychology and Neuroscience, De Crespigny Park, London, SE5 8AF UK; 4 Research Department of Behavioural Science and Health, Institute of Epidemiology & Health Care, University College London, Torrington Place, London, WC1E 7HB UK; 5 Department of Psychology, University of Alberta, P-355, Biological Sciences, Edmonton, AB T6G 2E9 Canada; 6 School of Health Sciences, Faculty of Biology, Medicine & Health, The University of Manchester, Oxford Road, Manchester, M13 9PL UK; 7 Department of Biostatistics and Health Informatics, King’s College London, Institute of Psychiatry, Psychology and Neuroscience, De Crespigny Park, London, SE5 8AF UK; 8 ImROC, Nottinghamshire Healthcare NHS Foundation Trust, Duncan Macmillan House, Porchester Road, Mapperley, Nottingham, NG3 6AA UK; 9 Department of Psychiatry, University of Toronto, Toronto, Ontario, M5T 1R8 Canada; 10 School of Allied Health, Human Services and Sport, College of Science, Health and Engineering, La Trobe University, Melbourne, VIC 110091 Australia; 11 Department of Special Needs Education, Ghent University, Henri Dunantlaan 2, 9000 Ghent, Belgium; 12 WHO Country Office in Bulgaria, World Health Organization, 15, Ivan Geshov Blvd, 1431 Sofia, Bulgaria; 13 Centre for Mental Health Care Development, Lublaňská 1730/21, 120 00 Praha 2, Prague, Czech Republic; 14 CORE: Copenhagen Research Center for Mental Health, Mental Health Centre Copenhagen, Kobenhavn, Denmark; 15 University of Tartu, Institute of Social Studies, Lossi 36, Tartu, Estonia; 16 Department of Psychiatry, Marseille Public Hospital, 147 Boulevard Baille, F-, 13005 Marseille, France; 17 Department of Psychiatry II, Ulm University, Günzburg, Germany; 18 Department of Mental Health and Addiction Services, Ausl Bologna, Bologna, Italy; 19 Department of Psychiatric Nursing, Graduate School of Medicine, University of Tokyo, Bunkyo-ku, Tokyo, 1130033 Japan; 20 Lentis Psychiatric Institute, Lentis Research, Groningen, the Netherlands; University of Groningen, Faculty of Behavioural and Social Sciences, Department of Clinical Psychology and Experimental Psychopathology, Groningen, The Netherlands; 21 Department of Health, Social and Welfare Studies, University of South-Eastern Norway, Postboks 235, 3603 Kongsberg, Norway; 22 Medical Social Work Department, Institute of Mental Health, 10 Buangkok View, 539747 Singapore, Singapore; 23 Excellence Center Srithanya Hospital, Department of Mental Health, 47 Talat Kwan, Mueang Nonthaburi District, Nonthaburi, 11000 Thailand; 24 Center for Psychiatric Rehabilitation, Boston University, Boston, MA 02215 USA; 25 Cardiff and Vale Recovery & Wellbeing College, Park Lodge, Whitchurch, Cardiff, CF14 7BL UK; 26 Drive Direction, 8 C Lambie Drive, Manukau, 2241 New Zealand; 27 Public Health Agency, Towerhill, Armagh, Northern Ireland BT61 9DR UK; 28 School of Health Sciences, Soroti University, P. O. Box 211, Soroti, Uganda; 29 Department of Social Work and Social Administration, The University of Hong Kong, Pokfulam Road, Hong Kong, China; 30 Special Education Faculty, ELTE University, Institute of Disability and Social Participation, Ecseri Street 3, Budapest, 1097 Hungary; 31 Community Health Organisation, Health Service Executive (HSE), Dublin, Ireland; 32 Recovery College Berne, University Hospital of Psychiatry and Psychotherapy, University Berne Psychiatric Services, Bolligenstrasse 60, 3000 Berne, Switzerland; 33 RECOLLECT Lived Experience Advisory Panel, London, UK; 34 WHO Collaborating Centre for Mental Health Research and Training, King’s College London, Institute of Psychiatry, Psychology and Neuroscience, De Crespigny Park, London, SE5 8AF UK; 35 Discovery College, Headspace Early Psychosis, Alfred Mental and Addiction Health, South East Melbourne, Melbourn, VIC Australia; 36 Recovery & Addiction cluster, Ghent University, Department of Special Needs Education, H. Dunantlaan 2, B-9000 Gent, Belgium; 37 Ontario Shores Centre for Mental Health Sciences, 700 Gordon Street, Whitby, ON L1N 5S9 Canada; 38 Canadian Mental Health Association (National), Toronto, ON M5T 2Z5 Canada; 39 Department of Health Sciences, Lund University, SE-221 00 Lund, Sweden; 40 Department of Research and Development, Division of Psychiatry, Region Skåne, Lund, Sweden; 41 Mind HK, 18 One Capital Place, Luard Rd, Wan Chai, Hong Kong; 42 MERITT Group. Institut de Recerca Sant Joan de Deu, Parc Sanitari Sant Joan de Deu, CIBERSAM, ISCIII, Sant Boi de Llobregat, 08330 Spain; 43 Unit of Population Health, University of Oulu, P.O.BOX 8000, FI-90014 University of Oulu, Oulu, Finland; 44 Faculty of Engineering, University of Nottingham, Nottingham, NG7 2RD UK; 45 Nord University, Faculty of Nursing and Health Sciences, Health and Community Participation Division, Postbox 474, 7801 Namsos, Norway

**Keywords:** Quality of life, Psychology

## Abstract

Recovery Colleges (RCs) are learning-based mental health recovery communities, located globally. However, evidence on RC effectiveness outside Western, educated, industrialised, rich, and democratic (WEIRD) countries is limited. This study aimed to evaluate associations between cultural characteristics and RC fidelity, to understand how culture impacts RC operation. Service managers from 169 RCs spanning 28 WEIRD and non-WEIRD countries assessed the fidelity using the RECOLLECT Fidelity Measure, developed based upon key RC operation components. Hofstede’s cultural dimension scores were entered as predictors in linear mixed-effects regression models, controlling for GDP spent on healthcare and Gini coefficient. Higher Individualism and Indulgence, and lower Uncertainty Avoidance were associated with higher fidelity, while Long-Term Orientation was a borderline negative predictor. RC operations were predominantly aligned with WEIRD cultures, highlighting the need to incorporate non-WEIRD cultural perspectives to enhance RCs’ global impact. Findings can inform the refinement and evaluation of mental health recovery interventions worldwide.

## Introduction

Recovery Colleges (RCs) are a relatively new learning-based mental health recovery support system offering information, social support and skill development for people with mental health symptoms, carers and staff. RCs were informed by the development of education centre and peer-run services for mental health recovery in the USA during the 1990s^1^. The first RC opened in England in 2009, and the approach has spread globally. Today RCs are in operation in 28 countries in Europe, Asia, Africa, North America and Oceania across different economic levels and cultural characteristics^2^. The service settings have also diversified, including primary and secondary care, non-governmental organisations, and education providers^3^. Key philosophies of RCs are co-production and adult education. Co-production means the involvement of lived experience and professional expertise in planning, designing, delivery, and quality assurance of the mental health courses for people with mental health symptoms, carers and staff^4^. Adult education is self-directed learning. Adults with a history of mental health symptoms engage with learning that is characterised as collaborative, strengths-based, person-centred, inclusive and community-focused^5,6^. These two key philosophies contribute to personal recovery, which is defined as living a purposeful and autonomous life despite the presence of mental health symptoms^1^. The co-production philosophy of RCs is underpinned by two conceptual shifts related to personal recovery from mental health symptoms: (a) the focus of care is on the person, rather than the symptoms, and (b) empowerment and quality of life are as important as symptom reduction^7^. Recovery is supported through social inclusion of the RC students, empowering them to have or increase social and/or economic roles. Current and former mental health service users, along with other stakeholders such as informal carers and clinical staff working at diverse settings, can enrol as students in a RC^7^. RCs provide educational and skill development courses for RC students to manage their own wellbeing, such as recovery planning, and mindfulness^8^. The courses are intended to support students to understand recovery, rebuild their life (e.g., improving sleep), develop life skills (e.g., moving towards life goals) and get more involved in a RC (e.g., qualification to be a peer trainer)^9^.

In order to understand how RCs work, a change model of RCs for service user students was developed from a systematised review (44 publications) and 33 stakeholder interviews^5^. Three steps were followed in this study. First, an initial change model was created based on 10 key publications inductively and collaboratively analysed by academic researchers and people with lived experience of mental health symptoms. Second, the initial model was refined through deductive analysis of 34 further publications. Third, the refined model was further refined through stakeholder interviews (*n* = 33). The interviewees included RC students who have also used secondary care mental health services, peer trainers, clinician trainers, RC managers. The finalised change model comprises mechanisms of action (how RCs work), and outcomes (impact of RCs). Four mechanisms of action were identified: (1) offering an empowering environment, (2) enabling different relationships, (3) facilitating personal growth, and (4) shifting the balance of power through co-production, reducing power differentials. Two categories of outcomes were identified: changes (1) in the student (e.g., self-confidence, self-management) and (2) in their life (e.g., having interests, social engagement).

The evidence base for the effectiveness and cost-effectiveness of RCs is still preliminary. Evidence syntheses including reviews about RCs report a lack of research published, and highlight methodological weaknesses^5,6,10,11^. However, the preliminary evidence is positive. A 2020 review appraising the impact of RCs reported student and staff benefits and initial cost-effectiveness^6^. RC student benefits include confidence, self-esteem, hope, healthier lifestyle, quality of life, and reduced stigma^5,6^. RC staff acquire skills and knowledge, leading to (a) changes in their attitudes towards co-production and service users, and (b) increased work motivation^10^. Regarding cost-effectiveness, an uncontrolled study comparing RC students who completed at least one course versus those who did not, identified that attending an RC was associated with fewer bed days, unintended hospitalisations, and community contacts over 18 months^12^. Similar results were reported from a service evaluation in the UK^13^. A cost-benefit analysis in Australia showed reduced emergency and inpatient service use with net savings of A$269 per student^14^. A 2022 thematic synthesis of qualitative evidence identified the positive RC impact on empowerment and inclusivity. However, methodological weaknesses such as sample selection biases were noted^11^.

One reason for under-developed RC evidence is unstandardised operation^4^. To address this problem, RC components were identified, and a fidelity measure was developed^4^. Fidelity refers to how much an intervention is carried out as planned^15^. Fidelity is especially important to multi-site interventions^15^, therefore is relevant to RC operation^16^. Fidelity components were identified through a multi-stage process: an initial list was developed through a systematised review (13 publications), followed by international expert consultation (*n* = 77) and interviews with RC managers in England (*n* = 10)^4^, and finally the list was refined through interviews with RC students, trainers and managers in England (*n* = 44). Those 12 identified fidelity components were categorised into seven nonmodifiable components (Valuing equality; Learning; Tailored to the student; Co-production; Social connectedness; Community focus; and Commitment to recovery) and five modifiable components (Available to all; Location; Distinctiveness of course content; Strengths-based; and Progressive)^4^. The resulting Recovery Colleges Characterisation and Testing (RECOLLECT) Fidelity Measure is a 12-item college manager-rated measure, with each item corresponding to one component. High fidelity indicates that the assessed RC is operated corresponding to what was regarded important in RC operation.

A knowledge gap exists in how culture impacts RC operation. One reason for this is that most RC research has been conducted in Western, educated, industrialised, rich and democratic (WEIRD) countries, lacking evidence from other countries^16,17^. To date, there have been six reviews about RCs published, and all included studies (*n* = 185), apart from one international study^18^, were from WEIRD countries: UK, Ireland, Australia, Canada, Italy, and USA (Supplementary Information 1)^4–6,10,19,20^. WEIRD countries account for only 12% of the world population, yet the majority of research samples (e.g., 96% of psychological samples) come from these countries. WEIRD countries share relatively similar cultural values such as individualistic, democratic, and greater freedom to satisfy the natural human needs to enjoy life^21^. The international study^18^ involved Hong Kong, Israel, Japan, Singapore, Sri Lanka and Singapore, but the findings were reported as an aggregate from the 22 participating counties (the remaining 16 countries were WEIRD countries). More recently, an England-Japan comparison of RC implementation was conducted^22^, highlighting a need for more cross-cultural studies in RC^23^. Taken together, very little RC evidence from non-WEIRD countries has been reported. Cross-cultural understanding of RCs can help reduce this knowledge gap.

Cultural adaptation is needed for RCs operating in non-WEIRD cultures, because most RC research has been conducted in WEIRD countries that share similar cultural values, which are often different from those in non-WEIRD countries^21^. Cultural adaptation is “the systematic modification of an evidence-based treatment…to consider language, culture, and context in such a way that it is compatible with the client’s cultural patterns, meaning, and values”^24^. The need for cultural adaptation in mental health treatment has been increasingly recognised^25^ due to factors such as migration, telecommunications and social media^26^. This applies not only at the global level but also at the national level, where evidence for people in cultural minorities remains to be identified^27^. Mental health cannot be fully understood without considering culture^28^, as all human experiences, including mental health, are shaped by cultural particulars^26^. A meta-analysis of randomised controlled trials identified that culturally-adapted treatment yielded greater mental health improvement than non-adapted active treatment^29^. Another meta-analysis reported that culturally-adapted treatment was nearly five times more likely to produce remission from mental health symptoms than non-culturally-adapted treatment^30^. The effect sizes of culturally-adapted treatment are moderate to large for people in non-WEIRD countries^25^. Cultural adaptation has been investigated for various mental health approaches, including cognitive behavioural therapy, and metacognitive therapy^25^. The extent to which cultural adaptation of RCs is needed when operated in non-WEIRD cultures is unknown.

To identify how RCs should be culturally adapted to non-WEIRD cultures, cultural characteristics related to RCs need to be identified. Hofstede’s cultural dimension theory^21^ is the most established quantitative framework in cross-cultural research^31^, defining culture as “the collective programming of the mind that distinguishes the members of one group or category of people from others”^32^. The cultural dimension theory proposes six cultural characteristics: Power Distance, Individualism, Success-Drivenness, Uncertainty Avoidance, Long-Term Orientation, and Indulgence. The meaning of each cultural characteristic is described in Table 1.

**Table 1:** Six cultural characteristics in the cultural dimension theory

Characteristic (Interpretation)	Meaning
Power Distance (high vs low)	A degree to which inequality and unequal distributions of power between parties are accepted.
Individualism (vs Collectivism)	A degree to which a society excepts individuals to be loosely tied to one another, and to take care of only themselves and their immediate family.
Success-Drivenness (vs Quality-Orientation)	A societal value for achievement, and material rewards for success (originally named ‘Masculinity’).
Uncertainty Avoidance (high vs low)	A degree to which individuals feel threatened by unknown situations, and try to avoid such situations.
Long-Term Orientation (vs Short-Term Orientation)	Values oriented towards future rewards, perseverance, and thrift, which are related to ‘saving’ as opposed to ‘spending’.
Indulgence (vs Self-Restrained)	Acceptance of relatively free gratification of basic and natural human needs to enjoy life.

These cultural characteristics have high relevance to RC operation. For example, one of the key RC philosophies, co-production may be relevant to Power Distance (e.g., between healthcare workers and service users) and Individualism, as inferred in our international RC study^2^. Co-production requires each individual to express their needs, which may have relevance to Power Distance, Individualism, Success-Drivenness, and Indulgence. These needs are individual, and may be challenging to articulate, which may be impacted by Uncertainty Avoidance. Finally, the RC focus on the person, instead of the symptom, may require Long-Term Orientation. However, these relationships have not been empirically evaluated.

Moreover, cross-cultural debates in RC research thus far have tended to be limited to broader categories (e.g., Asian collectivism vs Western individualism^2^). Among the same cultural characteristics, how much one characteristic is valued differs across cultures. For example, Thailand and Japan are both labelled as valuing collectivism in the West, but Japan is known to have more individualistic values than other Asian countries^33,34^. Empirical evaluation of the RC-culture relationships needs to consider these differences.

Our previous global survey^2^ identified preliminary evidence that cultural characteristics may influence fidelity ratings. Fidelity scores in RCs in Western countries were higher than those in non-Western countries. We speculated that one reason for the fidelity score gap might be cultural differences; however, an empirical evaluation remained to be performed. The current study extends this work in four ways: the first empirical evaluation, use of an established theoretical framework (Hofstede), inclusion of cultural covariates (% of GDP spent on healthcare and GINI coefficient of social inequality^35,36^), and consideration of between-country differences in valorisation of particular cultural characteristics (e.g., collectivism in Thailand versus Japan).

This study aimed to explore the relationships between cultural characteristics and fidelity in all currently-operating RCs internationally. RC inclusion criteria were targeting to support personal recovery, and prioritising co-production and adult learning.

Our research questions were;

1. Are there associations between cultural characteristics and the operational indicators of RC fidelity?, and
2. If there are, which cultural characteristics are associated with the operational indicators of RC fidelity?”

Addressing these research questions is intended to identify cultural impact on RC fidelity. Because RCs are in operation in many countries, identifying the cultural impact of RC operation will be useful to cross-cultural understanding of RC operation, and by extension, will have relevance to other recovery-oriented global innovations, such as mental health peer support work^37^. We recruited RCs that were currently in operation from 28 countries across different cultures (Table 2 for the participating WEIRD and non-WEIRD countries), and evaluated whether differences in cultural characteristics could predict variance in their fidelity scores. Mixed-effects linear regression models with a country-level random intercept were used to allow us to identify associations between the cultural characteristics and RC fidelity while accounting for variability between countries. No hypotheses were predefined due to the exploratory and inductive nature of the research^38^.

**Table 2:** Participating WEIRD and non-WEIRD countries (*n* = 28)

WEIRD	non-WEIRD
Australia	Hong Kong
Belgium	Japan
Bulgaria	Thailand
Canada	Uganda
Czechia	
Denmark	
England	
Estonia	
Finland	
France	
Germany	
Hungary	
Iceland	
Ireland	
Italy	
Jersey	
Netherlands	
New Zealand	
Northern Ireland	
Norway	
Scotland	
Spain	
Sweden	
Switzerland	
Wales	

Alphabetical order. *WEIRD* Western, educated, industrialised, rich, and democratic.

## Results

Unadjusted and adjusted associations between all cultural characteristics and RC fidelity scores are presented in Table 3. The percentage GDP spent on healthcare and Gini coefficients for each country were included as covariates in adjusted analyses.

**Table 3:** Associations between cultural characteristics and overall fidelity scores

	Unadjusted			Adjusted*		
	*N*	*β (95% CI)*	*p value*	*N*	*β (95% CI)*	*p value*
Power Distance	167	−0.03 (−0.08 to 0.01)	0.126	163	−0.04 (−0.08 to 0.01)	0.164
Individualism	167	**0.05 (0.01 to 0.08)**	0.004	163	**0.06 (0.02 to 0.10)**	0.002
Success-Drivenness	167	−0.01 (−0.03 to 0.02)	0.617	163	−0.01 (−0.04 to 0.02)	0.463
Uncertainty Avoidance	167	−**0.03 (**−**0.06 to** −**0.01)**	0.009	163	−**0.04 (**−**0.06 to** −**0.01)**	0.008
Long-Term Orientation	169	−0.02 (−0.05 to 0.01)	0.129	165	−**0.03 (**−**0.06 to** −**0.01)**	0.050
Indulgence	169	**0.04 (0.01 to 0.08)**	0.013	165	**0.05 (0.01 to 0.09)**	0.025

*Covariates: % GDP spent on healthcare, Gini coefficient. Bold indicates significant variables.

Hong Kong and New Zealand (2 RCs respectively) are excluded from the adjusted analysis as the Gini coefficients for these countries were not available from the World Bank. RCs in Uganda (*n* = 2) were included in the Long-Term Orientation and Indulgence only, as data for those two cultural characteristics were available.

Neither the percentage of GDP spent on healthcare and the Gini coefficients of included countries were associated with the fidelity scores, when (a) we examined simple associations between the percentage of GDP spent on healthcare and fidelity, and the Gini coefficients and fidelity, and (b) when we included those as covariates in the models with cultural characteristics.

**Fig. 1:**
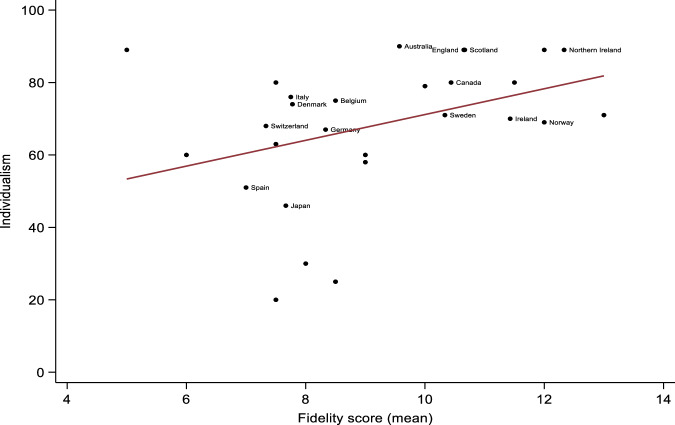
Association between Individualism and country-level Recovery College fidelity scores. Countries with data from <3 Recovery Colleges were blinded for anonymity purposes.

**Fig. 2:**
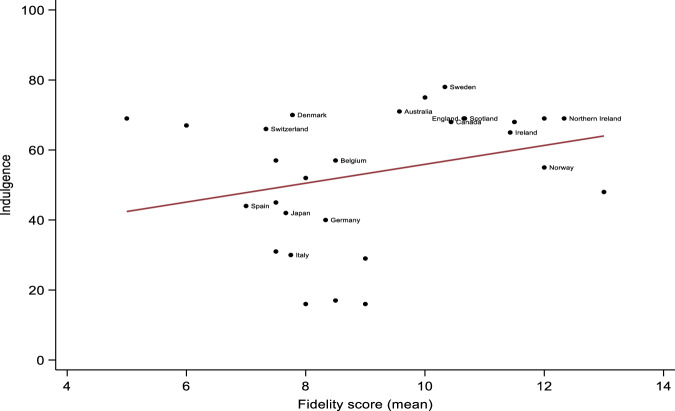
Association between Indulgence and country-level Recovery College fidelity scores. Countries with data from <3 Recovery Colleges were blinded for anonymity purposes.

**Fig. 3:**
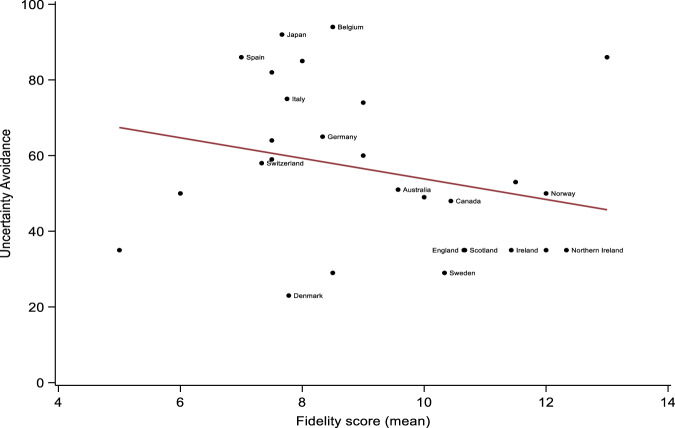
Association between Uncertainty Avoidance and country-level Recovery College fidelity scores. Countries with data from <3 Recovery Colleges were blinded for anonymity purposes.

Adjusted mixed-effects linear regressions showed that higher levels of Individualism (*β* = 0.06, 95% CI = 0.02 to 0.10, *p* = 0.002), and Indulgence (*β* = 0.05, 95% CI = 0.01 to 0.09, *p* = 0.025) were associated with higher fidelity scores. Conversely, higher levels of Uncertainty Avoidance were associated with lower fidelity scores (*β* = −0.04, 95% CI = −0.06 to −0.01, *p* = 0.008). Graphic representations of significant associations are provided in Figs. 1 to 3. There were no other significant associations between cultural characteristics and fidelity scores, although adjusted associations between Long-Term Orientation and fidelity could be considered ‘borderline’ (*β* = −0.03, 95% CI = −0.06 to −0.01, *p* = 0.050). This suggests that higher levels of Long-Term Orientation were associated with lower fidelity scores, once we accounted for the percentage of GDP spent on healthcare and the Gini coefficients of included countries. Power Distance (Unadjusted *β* = −0.03, 95% CI = −0.08 to 0.01, *p* = 0.126; Adjusted *β* = −0.04, 95% CI = −0.08 to 0.01, *p* = 0.164) and Success-Drivenness (Unadjusted *β* = −0.01, 95% CI = −0.03 to 0.02, *p* = 0.617; Adjusted *β* = −0.01, 95% CI = −0.04 to 0.02, *p* = 0.463) were not significantly associated with the RC fidelity scores.

**Answering research questions**

To answer our research questions;

1. Yes, there are associations between cultural characteristics and the operational indicators of RC fidelity.
2. Individualism and Indulgence are positively, and Uncertainty Avoidance is negatively associated with the operational indicators of RC fidelity. There are adjusted negative borderline associations between Long-Term Orientation and fidelity. Power Distance and Success-Drivenness are not associated with the fidelity.

This means that RCs operated in cultures that value individual needs (Individualism) and gratification of human needs (Indulgence) tend to receive positive impact on the fidelity of their RC operation. Conversely, RCs operated in cultures that try to avoid uncertain situations (Uncertainty Avoidance) tend to receive negative impact on the fidelity of their RC operation. Cultures that value patience and thriftiness (Long-Term Orientation) may have negative impact on the fidelity as this cultural characteristic was a borderline predictor.

## Discussion

In this global study, we found that higher levels of Individualism and Indulgence, and lower levels of Uncertainty Avoidance were associated with higher RC fidelity, with Long-Term Orientation being a borderline negative predictor. Cultures that prioritise needs of an individual and their immediate family (Individualism), that accept relatively free gratification of basic human needs to enjoy life (Indulgence), and that accept unknown situations (low Uncertainty Avoidance, i.e., Uncertainty Acceptance) tended to have higher fidelity. Moreover, cultures that focus on immediate results (low Long-Term Orientation, i.e., Short-Term Orientation) may have positive impact on the fidelity.

The results indicated that cultural characteristics typically associated with WEIRD countries predicted RC fidelity: high on Individualism and Indulgence, and low on Uncertainty Avoidance and Long-Term Orientation^32^. One interpretation is that the values and assumptions underpinning the RECOLLECT Fidelity Measure are more aligned with the values of WEIRD countries than non-WEIRD countries. RCs originate in, and are most established in, WEIRD countries^1^. It is possible that assumptions underpinning RCs reflect this origin. The key philosophies of RCs are co-production and adult education, which involve co-delivery of courses by peer and non-peer trainers who may model disagreement, and encourage students to express their individual needs so support can be tailored^39^. Disagreement and expression of individual needs are more accepted and indeed expected in individualistic and indulgent cultures than collectivistic and restrictive cultures^21^. Likewise, tolerance for individual and interpersonal differences is more afforded in uncertainty-accepting cultures than uncertainty-avoidant cultures^21^. Short-term-oriented cultures are more open to new ideas, which could facilitate the incorporation of new practices like RCs^21^. This may mean that the process of RC operation in non-WEIRD countries need to be understood more. For example, collectivistic cultures prioritise group harmony, therefore processes that are highlighted in co-production and adult education, such as disagreement and expression of individual needs, are in general not as accepted as in individualistic cultures^40^. Comparatively more people in collectivistic cultures may feel uncomfortable with these processes than those in individualistic cultures^41^. Moreover, people in collectivistic cultures in general tend to be more susceptible to shame towards mental health symptoms than those in individualistic cultures^42^. A better understanding of RC operation in non-WEIRD countries can inform how the current RC operation should be adjusted to maximise the global impact. The insights about cultural adaptation of RC operation can also help maximise the impact on minority culture groups within a country.

The findings of cultural influences on RC fidelity also raise concerns about ethnocentrism in valued outcomes. RC outcomes such as self-management and self-confidence^4^ may be more aligned with WEIRD cultures than non-WEIRD ones. Embedded assumptions in RCs about the importance of self-management may create a culture clash with student aspirations in collectivistic cultures to be seen as self-managing for not disclosing mental health symptoms to others^43^. This negative valorisation of an outcome, which is a focus of the RC, may lead students to disengage. Likewise, self-confidence is not as accepted in many non-WEIRD countries as in WEIRD countries^44^. Inclusion of more collective outcomes such as collective happiness^45^ where one feels happy when they know they are as happy as their peers instead of happier than their peers, may enhance cultural inclusivity of RC operation. Likewise, as a recent cross-cultural comparison of the RC advertisement texts reported, how RC students “learn together” (Collectivism) and experience “life-long learning” (Long-Term Orientation) may also need to be assessed to be inclusive of non-WEIRD cultures. These non-WEIRD outcomes can help people in RCs consider their wider community^27^, leading to a more holistic understanding of mental health and recovery^46,47^.

Regarding RC research, our results may highlight a need for a more culturally-informed measurement tool. Self-report measures are vulnerable to response bias, and respondents in WEIRD countries tend to demonstrate self-enhancement effects^42^. Self-enhancement is less encouraged in non-WEIRD cultures, as it could violate group harmony^21^. It is possible that even with the same level of fidelity, RC managers in WEIRD countries assessed their RC fidelity as high, whereas RC managers in non-WEIRD countries assessed theirs as low. If RC managers in non-WEIRD countries felt RC operation is oriented to WEIRD cultures, the self-enhancement of WEIRD managers might be even further accentuated^23^. Additionally, there was variance in fidelity scores among WEIRD countries. This variance may be attributed to factors such as (a) the RECOLLECT Fidelity Measure weighing all components equally,(b) variance in cultural characteristics among WEIRD countries, and (c) potential response biases among assessors. Future research should (a) evaluate the relative importance of each component, (b) compare cultures within WEIRD countries, and (c) involve individuals other than RC managers in the assessment of fidelity.

Our study has three implications for RC development and evaluation. First, the adult education focus needs to be inclusive of people from cultures that are not familiar with expression of, and adjustment for, individual needs. This can help include more people in cultures that value Collectivism and Self-Restraint (contrasting values to Individualism and Indulgence). Culturally appropriate approaches to supporting identification of needs, for example involving collective (e.g., family members) rather than individual decision-making, may be needed to ensure that changes in the student are positively valorised in their community. Second, the co-production focus needs to be interpreted through local cultural values. For example, recognising that higher external stigma (as opposed to internal stigma)^48^ experiences in non-WEIRD countries^44^ may require the peer trainer role to be modified to include individuals with lived experience as informal carers. The external presence of the peer trainer with lived experience can help reduce external stigma by supporting common humanity—awareness that it is not only them who experience mental health symptoms^49^. This can help include people oriented to cultures that value Long-Term Orientation as acknowledgement of difficulties in life is related to this value^50,51^. Moreover, whether co-production is a universally-accepted concept or not, needs to be discussed. In some non-WEIRD settings (e.g., Japanese and Thai cultures), clinical decision-making models developed in the West, have been challenged, because the models required individual service users to express their needs independently^52,53^. Cultural adaptation of RCs should include revisiting the key philosophies of RCs, to appraise the fitness to some non-WEIRD cultural characteristics. This can help ensure that good operation practice in one cultural context is not imposed on people in other cultural contexts. Epistemic injustice—people from marginalised groups are denied the chance to contribute to knowledge and interpret their own experiences—is increasingly gaining attention in mental health care^54^, and our findings can help manifest epistemic injustice in RCs. Finally, a culturally-adapted fidelity assessment approach is indicated. The advantages of using self-report measures, such as simplicity and timeliness, are helpful to RC research. However, the RECOLLECT Fidelity Measure can be refined by incorporating more non-WEIRD perspectives. This refinement includes addition/adjustment of items or wording, and culturally-adaptable translation that prioritises conceptual equivalence, instead of direct linguistic equivalence alone^55^. For example, the item about “Tailoring to students” is more relevant to uncertainty-accepting cultures, allowing individual differences^21^. Measures such as providing examples, and asking about previous student cases can help better understand about this item for more people in uncertainty-avoidant culture^56^.

Strengths of our study include it being the first global cross-cultural study of RCs in 28 countries, informing RC development globally. Service disparity for minority cultures is a global mental health concern, associated with poor service uptake, adverse mental health outcomes, and increased costs^25^. RCs are operating in 28 countries including low- and middle-income countries (LMICs). Our findings can inform cultural adaptation of RCs, helping to address service disparity. Additionally, the numbers of RCs in non-WEIRD countries, including LMICs (Table 4), suggest they may be in the early stages of RC implementation. Our findings could guide the initial steps towards scaling RCs in those countries. The establishment of more RCs in non-WEIRD countries would promote the inclusion of diverse cultures in RC operations worldwide. Although our study included all operational RCs globally, participation from non-WEIRD countries was limited, with only 15 RCs from four such countries represented. To better understand RC cultural adaptations in non-WEIRD contexts, a greater presence of non-WEIRD RCs is needed. Several study limitations can be identified. First, there are other cross-cultural frameworks that could have been used (e.g., tightness-looseness^57^). However, data for many of the 28 countries were not available, making meaningful comparisons problematic. Relatedly, critiques of Hofstede’s definition of culture^32^ include overgeneralisation such as treating nations as a cultural unit^58^ and under-emphasis on non-psychological cultural aspects such as socioeconomic and ecosocial factors^59,60^. To effectively inform the cultural adaptation of RC, these factors must be assessed using more in-depth approaches^61^. For example, community-based participatory research, which involves close collaboration with local communities, stakeholders, and cultural minority groups, is recommended to identify the most appropriate cultural adaptations^62^. Common research processes in WEIRD countries, such as interviews, can make people in non-WEIRD countries feel like ‘subjects’ thus may not capture authentic responses. Culturally appropriate processes, such as casually asking around in their natural environment (e.g., *Pagtatanong-tanong* in the Philipines^63^), can be more effective in eliciting genuine responses that are useful for cultural adaptations^64^. Moreover, the person-centred approach to recovery that RCs emphasise, may seem contradictory to our evaluation on cultures. However, the cultural dimension scores regard collective tendencies, instead of personal factors^32^. Therefore, our findings inform associations between cultural characteristics and RC operation. Second, although we included two relevant confounders in fully adjusted analysis, it is possible there were unmeasured confounders that may bias our results. We used these two confounders due to their relevance to mental health treatment resources^65^ and Hofstede’s index^35,36^, and the significant global variation in financial statuses for RC operations^2^. However, since no studies have directly examined the relationship between Hofstede’s index and mental health intervention fidelity, other country-level confounders, such as trust in government^66^, may also be relevant. Third, RCs with missing data for outcomes of interest or confounders were excluded. The uneven distribution of RCs across countries limits the robustness of the findings. Fourth, the survey was completed by service managers, therefore may not reflect the other people’s perspectives. Following the philosophy of RCs, the fidelity assessment should be done by RC students too. This raises the deeper issue of reducing fidelity to a quantitative score (as done with the RECOLLECT Fidelity Measure in this study), which may not capture many important operating characteristics, such as psychological safety and the impact of the built environment on student and trainer wellbeing. Future research should involve student assessment after addressing ethical concerns, with rigorous and reasonable sampling methods in each country or context (e.g., how to identify people who can assess an RC comprehensively). Consideration should also be given to developing more qualitative approaches to characterising fidelity, for example using the Impacts of Recovery Innovations (IMRI) framework^67^. Lastly, as cultures and practice change over time, cross-cultural understanding of RCs needs to be investigated periodically.

**Table 4:** STROBE guidelines

	Item No.	Recommendation	Page No.	Relevant text from manuscript
**Title and abstract**	1	(a) Indicate the study’s design with a commonly used term in the title or the abstract	1, 5	“associations” in the title and abstract. “linear mixed-effects regression models“ in the abstract.
		(b) Provide in the abstract an informative and balanced summary of what was done and what was found	5	What was done: “RC fidelity data were collected from 169 of all 221 RCs currently operating, spanning 28 WEIRD and non-WEIRD countries. Hofstede’s cultural dimension scores were entered as predictors in linear mixed-effects regression models, controlling for GDP percentage spent on healthcare and Gini coefficient.” What was found: “Higher Individualism (β = 0.06) and Indulgence (β = 0.05), as well as lower Uncertainty Avoidance (β = −0.04) were associated with higher RC fidelity with Long-Term Orientation being a borderline negative predictor of the fidelity (β = −0.03).”
**Introduction**				
Background/rationale	2	Explain the scientific background and rationale for the investigation being reported	6–8	The scientific background: Despite RCs located in many countries including both WEIRD countries and non-WEIRD countries, evidence base for how RCs can help mental health recovery is oriented to WEIRD countries only. There are six reviews about RCs, and all included studies apart from one international study were from WEIRD countries (185 included studies in total). Rationale: Cross-cultural understanding of RCs remains to be developed. RCs are in operation in non-WEIRD countries including low- and middle-income countries.
Objectives	3	State specific objectives, including any prespecified hypotheses	8	“This study aimed to explore the relationships between cultural characteristics and fidelity in all currently-operating RCs internationally. RC inclusion criteria were targeting to support personal recovery, and prioritising co-production and adult learning. Our research questions were; 1. Are there associations between cultural characteristics and the operational indicators of RC fidelity?, and 2. If there are, which cultural characteristics are associated with the operational indicators of RC fidelity?” Addressing these research questions is intended to identify cultural impact on RC fidelity. Because RCs are in operation in many countries, identifying the cultural impact of RC operation will be useful to cross-cultural understanding of RC operation, and by extension, will have relevance to other recovery-oriented global innovations, such as mental health peer support work^37^. We recruited RCs that were currently in operation from 28 countries across different cultures (Table 2 for the participating WEIRD and non-WEIRD countries), and evaluated whether differences in cultural characteristics could predict variance in their fidelity scores. Mixed-effects linear regression models with a country-level random intercept were used to allow us to identify associations between the cultural characteristics and RC fidelity while accounting for variability between countries. No hypotheses were predefined due to the exploratory and inductive nature of the research^38^.
**Methods**				
Study design	4	Present key elements of study design early in the paper	1, 5, 8 and 12	Noted above, in 1(a) and 3. Additionally, “We conducted a cross-sectional, observational survey in two rounds: first of all RCs in England (“England survey”)^3^, then of RCs in all other countries (“international survey”)^2^. Approval was obtained from King’s College London Research Ethics Psychiatry Nursing and Midwifery Subcommittee on 09/02/22 (MRA-21/22-28685). All participants provided written informed consent prior to completing the survey. The study was conducted as part of the RECOLLECT programme^16^. RECOLLECT is a five-year (2020-2025) National Institute for Health and Care Research (NIHR)-funded research programme exploring the effectiveness and cost-effectiveness of RCs^16^.”
Setting	5	Describe the setting, locations, and relevant dates, including periods of recruitment, exposure, follow-up, and data collection	12	“We included all RCs whose managers completed the RECOLLECT Fidelity Measure between August and October 2021 for the England survey, and between February and October 2022 for the international survey.
Participants	6	(a) Cross-sectional study—Give the eligibility criteria, and the sources and methods of selection of participants	13 and Supplementary Information 2	“Because not all RCs named themselves a “Recovery College” (e.g., “Recovery Academy”, “Recovery School”), we included any currently active services that met three criteria, informed by the key RC components^4^. The criteria were: (a) targeting to support personal recovery; (b) prioritising co-production and (c) adult learning, and were confirmed by the service managers. Full details are reported elsewhere^2^, and presented in Supplementary Information 2.”
Variables	7	Clearly define all outcomes, exposures, predictors, potential confounders, and effect modifiers. Give diagnostic criteria, if applicable	14	Outcome variable, predictor variables, and confounder variables are defined.
Data sources/ measurement	8	For each variable of interest, give sources of data and details of methods of assessment (measurement). Describe comparability of assessment methods if there is more than one group	13-14	“Fidelity was measured using the seven nonmodifiable components of the RECOLLECT Fidelity Measure^4^, completed by the manager of each RC.” Predictor variable: “Data for the cultural characteristics were obtained from Hofstede^70^…. The data were collected using the Value Survey Module 2013” Confounder variables: “Two confounder variables were included in the fully adjusted analyses, as relevant to mental health treatment resources^65^ and Hofstede’s index^35,36^, as well as the significant global variation in financial statuses for RC operations^2^. The percentage of GDP spent on health is the amount spent on healthcare relative to the economy size, calculated by the total health expenditure divided by GDP^72^. The Gini coefficient for each country indicates the income inequality within a nation, expressed from 0 (perfect equality) to 1 (maximum inequality), obtained from the World Bank^73^.”
Bias	9	Describe any efforts to address potential sources of bias	14	“Fidelity scores were summarised as medians and interquartile ranges where possible (i.e. for countries which provided fidelity data for multiple RCs). In order to examine unadjusted and adjusted associations between each cultural characteristic (country-level) and fidelity scores (college-level), we used mixed-effects linear regression models with a country-level random intercept in order to account for variability between countries. Adjusted associations included the percentage of GDP spent on healthcare and the Gini coefficient for each country as potential confounders. Uganda were missing data for Power Distance, Individualism, Success-Drivenness, and Uncertainty Avoidance, and was therefore excluded from analyses involving these cultural predictors. Gini coefficients for Hong Kong and New Zealand were unavailable from the World Bank due to high costs (Personal communication on 28 April 2023, The World Bank, Development Economics Data Group): these two countries were omitted from adjusted mixed-effects linear regression models.”
Study size	10	Explain how the study size was arrived at	12-13	“We included all RCs whose managers completed the RECOLLECT Fidelity Measure between August and October 2021 for the England survey, and between February and October 2022 for the international survey.”Three steps were followed for both surveys: (1) Developing RC inclusion criteria, (2) Identifying and approaching potentially eligible RCs, and (3) Disseminating and collecting the survey.”
Quantitative variables	11	Explain how quantitative variables were handled in the analyses. If applicable, describe which groupings were chosen and why	13-14	All quantitative variables were handled as continuous variables. This is detailed for Fidelity, Cultural Characteristics, and confounder variables.
Statistical methods	12	(a) Describe all statistical methods, including those used to control for confounding	14-15	Mixed-effects linear regression models - efforts to control for confounding are described in the Statistical analysis section.
		(b) Describe any methods used to examine subgroups and interactions	N/A	The examination of subgroups or interactions was not applicable in this study
		(c) Explain how missing data were addressed	14-15 and Table 3	Complete case analysis - countries were omitted from analyses if data were missing. Imputation was not appropriate.
		(d) Cross-sectional study—If applicable, describe analytical methods taking account of sampling strategy	N/A	A complex sampling strategy was not used in this study, so we did not need to adjust for this.
		(e) Describe any sensitivity analyses	N/A	No sensitivity analyses were performed.
**Results**				
Participants	13	(a) Report numbers of individuals at each stage of study—e.g. numbers potentially eligible, examined for eligibility, confirmed eligible, included in the study, completing follow-up, and analysed	14 and Table 5	“The surveys were completed by 169 (76%) RC managers from 28 countries, with more than 55,000 students attending in total. A description of the sample and summaries of variables of interest are provided in Table 5.”
		(b) Give reasons for non-participation at each stage	14–15 and Table 5	Statistical analysis section and Table 5 provide sample sizes that indicate non-participation in analyses.
		(c) Consider use of a flow diagram	N/A	
Descriptive data	14	(a) Give characteristics of study participants (ego demographic, clinical, social) and information on exposures and potential confounders	Table 5	
		(b) Indicate number of participants with missing data for each variable of interest	Table 3	The number of colleges with missing data for certain variables are provided in Table 3 (e.g., Gini coefficient missing for New Zealand = 2 RCs).
		(c) Cohort study—Summarise follow-up time (e.g., average and total amount)	N/A	
Outcome data	15	*Cross-sectional study—Report numbers of outcome events or summary measures*	Table 3	
Main results	16	(a) Give unadjusted estimates and, if applicable, confounder-adjusted estimates and their precision (ego, 95% confidence interval). Make clear which confounders were adjusted for and why they were included	Table 3	Unadjusted and adjusted estimates and 95% CI are provided. Table 3 also reports the results of all outcomes, both significant and no significant associations. Clear list of covariates in table footnote.
		(b) Report category boundaries when continuous variables were categorized	N/A	No continuous variables were categorised.
		(c) If relevant, consider translating estimates of relative risk into absolute risk for a meaningful time period	N/A	We do not report relative risk.
Other analyses	17	Report other analyses done—e.g. analyses of subgroups and interactions, and sensitivity analyses	None	
**Discussion**				
Key results	18	Summarise key results with reference to study objectives	10	“In this global study, we found that higher levels of Individualism and Indulgence, and lower levels of Uncertainty Avoidance were associated with higher RC fidelity scores, with Long-Term Orientation being a borderline negative predictor.”
Limitations	19	Discuss limitations of the study, taking into account sources of potential bias or imprecision. Discuss both direction and magnitude of any potential bias	11-12	“Strengths of our study include it being the first global cross-cultural study of RCs in 28 countries, informing RC development globally. Service disparity for minority cultures is a global mental health concern, associated with poor service uptake, adverse mental health outcomes, and increased costs^25^. RCs are operating in 28 countries including low- and middle-income countries (LMICs). Our findings can inform cultural adaptation of RCs, helping to address service disparity. Additionally, the numbers of RCs in non-WEIRD countries, including LMICs (Table 4), suggest they may be in the early stages of RC implementation. Our findings could guide the initial steps towards scaling RCs in those countries. The establishment of more RCs in non-WEIRD countries would promote the inclusion of diverse cultures in RC operations worldwide. Although our study included all operational RCs globally, participation from non-WEIRD countries was limited, with only 15 RCs from four such countries represented. To better understand RC cultural adaptations in non-WEIRD contexts, a greater presence of non-WEIRD RCs is needed. Several study limitations can be identified. First, there are other cross-cultural frameworks that could have been used (e.g., tightness-looseness^57^). However, data for many of the 28 countries were not available, making meaningful comparisons problematic. Relatedly, critiques of Hofstede’s definition of culture^32^ include overgeneralisation such as treating nations as a cultural unit^58^ and under-emphasis on non-psychological cultural aspects such as socioeconomic and ecosocial factors^59,60^. To effectively inform the cultural adaptation of RC, these factors must be assessed using more in-depth approaches^61^. For example, community-based participatory research, which involves close collaboration with local communities, stakeholders, and cultural minority groups, is recommended to identify the most appropriate cultural adaptations^62^. Common research processes in WEIRD countries, such as interviews, can make people in non-WEIRD countries feel like ‘subjects’ thus may not capture authentic responses. Culturally appropriate processes, such as casually asking around in their natural environment (e.g., *Pagtatanong-tanong* in the Philipines^63^), can be more effective in eliciting genuine responses that are useful for cultural adaptations^64^. Moreover, the person-centred approach to recovery that RCs emphasise, may seem contradictory to our evaluation on cultures. However, the cultural dimension scores regard collective tendencies, instead of personal factors^32^. Therefore, our findings inform associations between cultural characteristics and RC operation. Second, although we included two relevant confounders in fully adjusted analysis, it is possible there were unmeasured confounders that may bias our results. We used these two confounders due to their relevance to mental health treatment resources^65^ and Hofstede’s index^35,36^, and the significant global variation in financial statuses for RC operations^2^. However, since no studies have directly examined the relationship between Hofstede’s index and mental health intervention fidelity, other country-level confounders, such as trust in government^66^, may also be relevant. Third, RCs with missing data for outcomes of interest or confounders were excluded. The uneven distribution of RCs across countries limits the robustness of the findings. Fourth, the survey was completed by service managers, therefore may not reflect the other people’s perspectives. Following the philosophy of RCs, the fidelity assessment should be done by RC students too. This raises the deeper issue of reducing fidelity to a quantitative score (as done with the RECOLLECT Fidelity Measure in this study), which may not capture many important operating characteristics, such as psychological safety and the impact of the built environment on student and trainer wellbeing. Future research should involve student assessment after addressing ethical concerns, with rigorous and reasonable sampling methods in each country or context (e.g., how to identify people who can assess an RC comprehensively). Consideration should also be given to developing more qualitative approaches to characterising fidelity, for example using the Impacts of Recovery Innovations (IMRI) framework^67^. Lastly, as cultures and practice change over time, cross-cultural understanding of RCs needs to be investigated periodically.”
Interpretation	20	Give a cautious overall interpretation of results considering objectives, limitations, multiplicity of analyses, results from similar studies, and other relevant evidence	10-12	From “The results indicated that characteristics typically associated with WEIRD countries predicted RC fidelity.” To “Lastly, as cultures and practice change over time, cross-cultural understanding of RCs needs to be investigated periodically.”
Generalisability	21	Discuss the generalisability (external validity) of the study results	10	From “The results indicated that characteristics typically associated with WEIRD countries predicted RC fidelity.” To “leading to a more holistic understanding of mental health and recovery^46,47^.”
**Other information**				
Funding	22	Give the source of funding and the role of the funders for the present study and, if applicable, for the original study on which the present article is based	12	“The study was conducted as part of the RECOLLECT programme^16^. RECOLLECT is a five-year (2020-2025) National Institute for Health and Care Research (NIHR)-funded research programme exploring the effectiveness and cost-effectiveness of RCs^16^.”

RCs are a relatively new approach to mental health recovery. Currently, RC research and operations remain focused on WEIRD contexts, with non-WEIRD cultures being under-represented. However, the extent to which RC research and operations are WEIRD-focused had not been empirically assessed until now. Our findings identified Individualism, Indulgence and Uncertainty Avoidance as significant predictors, and Long-Term Orientation as a borderline predictor of fidelity. These cultural characteristics can serve as the first step towards an empirically informed cultural adaption of RCs and other recovery-oriented complex interventions, maximising their global health impact.

## Methods

### Study design

We conducted a cross-sectional, observational survey in two rounds: first of all RCs in England (“England survey”)^3^, then of RCs in all other countries (“international survey”)^2^. Approval was obtained from King’s College London Research Ethics Psychiatry Nursing and Midwifery Subcommittee on 09/02/22 (MRA-21/22-28685). All participants provided written informed consent prior to completing the survey. The study was conducted as part of the RECOLLECT programme^16^. RECOLLECT is a five-year (2020-2025) National Institute for Health and Care Research (NIHR)-funded research programme exploring the effectiveness and cost-effectiveness of RCs^16^.

We included all RCs whose managers completed the RECOLLECT Fidelity Measure between August and October 2021 for the England survey, and between February and October 2022 for the international survey. In total, 28 countries from Africa, Asia, Europe, North America, and Oceania, were included. This study is a post-hoc analysis of data obtained from the England^3^ and international surveys^2^, targeting the cultural aspects of RCs. The STROBE guidelines were followed (Table 4).

### Procedures

Three steps were followed for both surveys: (1) Developing RC inclusion criteria, (2) Identifying and approaching potentially eligible RCs, and (3) Disseminating and collecting the survey.

(1) Developing RC inclusion criteria: Because not all RCs named themselves a “Recovery College” (e.g., “Recovery Academy”, “Recovery School”), we included any currently active services that met three criteria, informed by the key RC components^4^. The criteria were (a) targeting to support personal recovery; (b) prioritising co-production and (c) adult learning, and were confirmed by the service managers. Full details are reported elsewhere^2^, and presented in Supplementary Information 2.

(2) Identifying and approaching potentially eligible RCs: For the England survey, four approaches were undertaken to identify potentially eligible RCs in June and July 2021: (a) online searches; (b) consultation with RC national leaders and recovery networks such as ImROC (imroc.org); (c) snowball sampling, and (d) telephone calls to potential host charities and mental health service providers. The research team approached the identified to ensure whether the service met the inclusion criteria.

For the international survey, first, RC operating countries were identified. An initial list of countries was created through (a) a RC international survey^18^, (b) enquires to existing RC organisations, (c) expert consultation with 23 recovery experts, and (d) communications with our collaborators in countries where similar services were available (e.g., peer support). Second, we identified country leads in the listed countries using networks developed in the previous process. Each country lead searched the literature in their local language to identify RCs in their country. Third, country leads discussed with the service managers to ascertain whether their service met the inclusion criteria. Snowball sampling was used asking whether those managers knew of any other services that might meet the criteria.

(3) Disseminating and collecting the survey: For the England survey, a pilot survey was created using the Checklist for Reporting Results of Internet E-Surveys (CHERRIES) guidelines^68^, revised based on expert review, and responded by two RC managers. No changes were made to the fidelity measure^3^. The eligible service managers were asked to complete the survey on Qualtrics.

The international survey was adapted from the England survey by adjusting phrases (e.g., “NHS services” to “health services”). The adapted survey was piloted by three RC experts in Australia, Canada, and Japan. No changes were made to the fidelity measure^2^. The final version was sent out by country leads to RC managers in their countries in two forms, Qualtrics and Microsoft Word. In countries where English was not commonly used and multiple RCs were in operation, the country leads were asked to translate the survey into their local language using Microsoft Word. The translated version was communicated to RC managers in an online or in-person meeting, or a written document. The accuracy of each translation was checked by a second translator. Seven language versions were developed^69^: Danish, Dutch, French, German, Japanese, Mandarin-Chinese, and Norwegian. Completed Qualtrics survey responses were directly accessible by the research team. Completed Microsoft Word survey responses were encrypted and emailed to the research team by the RC manager or the country lead. The research team then input the data to the Qualtrics. Data from the England survey and the international survey were integrated. No financial incentives were offered in either survey.

### Eligible RCs

For the England survey, 134 services were identified as potentially eligible, of which 88 (66%) were confirmed to meet the inclusion criteria. 46 services were excluded, most commonly due to being non-contactable and deemed no longer operating (*n* = 20). Full details of exclusion reasons are reported elsewhere^3^.

For the international survey, 49 countries were included in the initial list as a potentially RC operating country. After expert consultation and country leads’ searches, the final list was made involving 30 countries with 211 potential RCs identified. Country leads contacted all potential RCs in their country. Two countries and 78 potential RCs were removed for not meeting the RC inclusion criteria. The most common reason for exclusion was non-contactable and deemed no longer operating (*n* = 22). Full details of exclusion reasons are reported elsewhere^2^.

### Participating RCs

The two surveys indicated that in 2021/2022 there were 221 RCs in 28 countries across Europe, Asia, Africa, North America and Oceania, with none in South America or Antarctica. The surveys were completed by 169 (76%) RC managers from 28 countries, with more than 55,000 students attending in total^2,3^. A description of the sample and summaries of variables of interest are provided in Table 5.

**Table 5:** Sample characteristics

Country (*n* = 28)	Recovery College (*n* = 169/221: responded/total)	% GDP spent on healthcare	Gini coefficient	Fidelity scores (median (IQR))	Power Distance	Individualism	Success-Drivenness	Uncertainty Avoidance	Long-Term Orientation	Indulgence
*Africa (n* = *1)*	*2/2*									
Uganda	2/2	3.8	42.8	Blinded	-	-	-	-	24	52
*Asia (n* = *3)*	*13/15*									
Hong Kong	2/2	5.3	-	Blinded	68	25	57	29	61	17
Japan	9/11	10.7	32.9	7 (6 to 10)	54	46	95	92	88	42
Thailand	2/2	3.8	36.4	Blinded	64	20	34	64	32	45
*Europe (n* = *21)*	*129/170*									
Belgium	10/14	10.7	27.2	8.5 (8 to 10)	65	75	54	94	82	57
Bulgaria	1/1	7.1	41.3	Blinded	70	30	40	85	69	16
Czechia	1/1	7.8	25.0	Blinded	57	58	57	74	70	29
Denmark	9/9	10.0	28.2	8 (6 to 9)	18	74	16	23	35	70
England	63/88	10.1	35.1	11 (9 to 13)	35	89	66	35	51	69
Estonia	2/2	6.7	30.3	Blinded	40	60	30	60	82	16
Finland	2/2	9.1	27.3	Blinded	33	63	26	59	38	57
France	1/1	11.1	32.4	Blinded	68	71	43	86	63	48
Germany	3/3	11.7	31.7	9 (6 to 10)	35	67	66	65	83	40
Hungary	2/3	6.3	29.6	Blinded	46	80	88	82	58	31
Iceland	1/1	8.6	26.1	Blinded	30	60	10	50	28	67
Ireland	7/11	6.7	30.6	11 (10 to 13)	28	70	68	35	24	65
Italy	4/4	8.7	35.2	7.5 (5 to 10.5)	50	76	70	75	61	30
Jersey^a^	1/1	10.1	35.1	Blinded	35	89	66	35	51	69
Netherlands	2/2	10.1	28.1	Blinded	38	80	14	53	67	68
Northern Ireland	3/4	10.1	35.1	13 (10 to 14)	35	89	66	35	51	69
Norway	4/5	10.5	27.6	12.5 (11 to 13)	31	69	8	50	35	55
Scotland	3/3	10.1	35.1	11 (9 to 12)	35	89	66	35	51	69
Spain	3/6	9.1	34.7	6 (5 to 10)	57	51	42	86	48	44
Sweden	3/3	10.9	30.0	11 (6 to 14)	31	71	5	29	53	78
Switzerland	3/4	11.3	33.1	8 (5 to 9)	34	68	70	58	74	66
Wales	1/2	10.1	35.1	Blinded	35	89	66	35	51	69
*Oceania (n* = *2)*	*9/11*									
Australia	7/9	9.9	34.3	10 (6 to 13)	38	90	61	51	21	71
New Zealand	2/2	9.7	-	Blinded	22	79	58	49	33	75
*North America (n* = *1)*	*16/23*									
Canada	16/23	10.8	33.3	10.5 (9 to 12)	39	80	52	19	36	68

^a^Jersey is a self-governing dependency of the UK and was not included in the overall number of countries, and not included in the analysis.

Most participating RCs (159 RCs; 94%) were located in WEIRD countries.

### Fidelity scores (Outcome variable)

Fidelity was measured using the seven nonmodifiable components of the RECOLLECT Fidelity Measure^4^, completed by the manager of each RC. The seven components are (1) equality, (2) adult learning, (3) tailoring to the student, (4) co-production, (5) social connectedness, (6) community focus, and (7) commitment to recovery. Responses are on a three-point ordinal scale from 0 (low fidelity) to 2^4^. The fidelity score is the sum of these seven items, ranging from 0 (low fidelity) to 14. The measure satisfies scaling assumptions, demonstrating adequate internal consistency (0.72), test–retest reliability (0.60), content validity and discriminant validity^4^.

### Cultural characteristics (Predictor variables)

Data for the cultural characteristics were obtained from Hofstede^70^. This dataset provides scores for the six cultural dimensions of 111 countries. The data were collected using the Value Survey Module 2013, a 24-item self-report measure responded on a five-point Likert scale from 1 to 5^71^. Each dimension score is calculated using mean scores of four different items and index formulas in the manual, presented from 0 (low) to 100^71^.

### Confounder variables

Two confounder variables were included in the fully adjusted analyses, as relevant to mental health treatment resources^65^ and Hofstede’s index^35,36^, as well as the significant global variation in financial statuses for RC operations^2^. The percentage of GDP spent on health is the amount spent on healthcare relative to the economy size, calculated by the total health expenditure divided by GDP^72^. The Gini coefficient for each country indicates the income inequality within a nation, expressed from 0 (perfect equality) to 1 (maximum inequality), obtained from the World Bank^73^.

### Statistical analysis

Fidelity scores were summarised as medians and interquartile ranges where possible (i.e. for countries which provided fidelity data for multiple RCs). In order to examine unadjusted and adjusted associations between each cultural characteristic (country-level) and fidelity scores (college-level), we used mixed-effects linear regression models with a country-level random intercept in order to account for variability between countries. Adjusted associations included the percentage of GDP spent on healthcare and the Gini coefficient for each country as potential confounders. Uganda were missing data for Power Distance, Individualism, Success-Drivenness, and Uncertainty Avoidance, and was therefore excluded from analyses involving these cultural predictors. Gini coefficients for Hong Kong and New Zealand were unavailable from the World Bank due to high costs (Personal communication on 28 April 2023, The World Bank, Development Economics Data Group): these two countries were omitted from adjusted mixed-effects linear regression models. All analyses were conducted using STATA 17.0 (StataCorp LLP, College Station, TX).

## Supplementary information

Supplementary information

## Data Availability

The data that support the findings of this study are available on request from the corresponding author. The data are not publicly available due to it containing identifiable information about RCs.

## Code availability

No code was used in this study.
