# Supplementary material for: 28-country global study on associations between cultural characteristics and Recovery College fidelity
Source: Npj Ment Health Res. 2024 Oct 8;3:46. doi: 10.1038/s44184-024-00092-9 (PMC11461659; doi:10.1038/s44184-024-00092-9)
Supplement: Supplementary file 1 — Supplementary information [file 44184_2024_92_MOESM1_ESM.pdf]

Supplementary Information 1. Included studies in six reviews about Recovery Colleges (retrieved on MEDLINE, Embase, Cochrane, CINAHL, and PsycINFO on 30 January 2023).

Included sources in Bester KL, McGlade A, Darragh E. Is co-production working well in recovery colleges? Emergent themes from a systematic narrative review. *The Journal of Mental Health Training, Education and Practice* 2022; **17**(1): 48-60.

	Reference	Country
1	Cameron J, Hart A, Brooker S, Neale P, Reardon M. Collaboration in the design and delivery of a mental health Recovery College course: experiences of students and tutors. *J Ment Health* 2018; **27**(4): 374-81.	UK
2	Crowther A, Taylor A, Toney R, et al. The impact of Recovery Colleges on mental health staff, services and society. *Epidemiology and Psychiatric Sciences* 2019; **28**(5): 481-8.	UK
3	Dalgarno M, Oates J. The meaning of co-production for clinicians: An exploratory case study of Practitioner Trainers in one Recovery College. *Journal of Psychiatric and Mental Health Nursing* 2018; **25**(5-6): 349-57.	UK
4	McGregor J, Repper J, Brown H. “The college is so different from anything I have done”. A study of the characteristics of Nottingham Recovery College. *The Journal of Mental Health Training, Education and Practice* 2014; **9**(1): 3-15.	UK
5	Meddings S, Guglietti S, Lambe H, Byrne D. Student perspectives: recovery college experience. *Mental Health and Social Inclusion* 2014; **18**(3): 142-50.	UK
6	Meddings S, Byrne D, Barnicoat S, Campbell E, Locks L. Co-delivered and co-produced: creating a recovery college in partnership. *The Journal of Mental Health Training, Education and Practice* 2014; **9**(1): 16-25	UK
7	Perkins AM, Ridler JH, Hammond L, Davies S, Hackmann C. Impacts of attending recovery colleges on NHS staff. *Mental Health and Social Inclusion* 2017; **21**(1): 18-24.	UK
8	Sommer J, Gill K, Stein-Parbury J. Walking side-by-side: Recovery Colleges revolutionising mental health care. *Mental Health and Social Inclusion* 2018; **22**(1): 18-26.	Australia
9	Windsor L, Roberts G, Dieppe P. Recovery Colleges – safe, stimulating and empowering. *Mental Health and Social Inclusion* 2017; **21**(5): 280-8.	UK
10	Zabel E, Donegan G, Lawrence K, French P. Exploring the impact of the recovery academy: a qualitative study of Recovery College experiences. *The Journal of Mental Health Training, Education and Practice* 2016; **11**(3): 162-71.	UK

Included sources in Lin E, Harris H, Black G, et al. Evaluating recovery colleges: a co-created scoping review. *Journal of Mental Health* 2022: 1-22.

	Reference	Country
1	Bourne P, Meddings S, Whittington A. An evaluation of service use outcomes in a Recovery College. *Journal of Mental Health* 2018; **27**(4): 359-66.	UK
2	Burhouse A, Rowland M, Marie Niman H, et al. Coaching for recovery: a quality improvement project in mental healthcare. *BMJ Quality Improvement Reports* 2015; **4**(1): u206576.w2641.	UK
3	Cameron J, Hart A, Brooker S, Neale P, Reardon M. Collaboration in the design and delivery of a mental health Recovery College course: experiences of students and tutors. *J Ment Health* 2018; **27**(4): 374-81.	UK

4	Critchley A, Dokter D, Odell-Miller H, Power N, Sandford S. Starting from Scratch: Co-production with dramatherapy in a Recovery College. *Dramatherapy* 2019; **40**(2): 63-80.	UK
5	Cronin P, Stein-Parbury J, Sommer J, Gill KH. What about value for money? A cost benefit analysis of the South Eastern Sydney Recovery and Wellbeing College. *Journal of Mental Health* 2021: 1-8.	Australia
6	Dunn EC, Sally Rogers E, Hutchinson DS, et al. Results of an Innovative University-based Recovery Education Program for Adults with Psychiatric Disabilities. *Administration and Policy in Mental Health and Mental Health Services Research* 2008; **35**(5): 357-69.	United States
7	Durbin A, Nisenbaum R, Wang R, Hwang SW, Kozloff N, Stergiopoulos V. Recovery Education for Adults Transitioning From Homelessness: A Longitudinal Outcome Evaluation. *Frontiers in Psychiatry* 2021; **12**.	Canada
8	Ebrahim S, Glascott A, Mayer H, Gair E. Recovery Colleges; how effective are they? *The Journal of Mental Health Training, Education and Practice* 2018; **13**(4): 209-18.	UK
9	Frayn E, Duke J, Smith H, Wayne P, Roberts G. A voyage of discovery: setting up a recovery college in a secure setting. *Mental Health and Social Inclusion* 2016; **20**(1): 29-35.	UK
10	Hall T, Jordan HL, Reifels L, et al. A Process and Intermediate Outcomes Evaluation of an Australian Recovery College. *Journal of Recovery in Mental Health* 2018; **1**(3): 7-20.	Australia
11	Harper L, McKeown M. Why make the effort? Exploring recovery college engagement. *Mental Health and Social Inclusion* 2018; **22**(1): 27-33.	UK
12	Hopkins L, Foster A, Nikitin L. The process of establishing Discovery College in Melbourne. *Mental Health and Social Inclusion* 2018; **22**(4): 187-94.	Australia
13	Hopkins L, Pedwell G, Lee S. Educational outcomes of Discovery College participation for young people. *Mental Health and Social Inclusion* 2018; **22**(4): 195-202.	Australia
14	Hopkins L, Foster A, Belmore S, Anderson S, Wiseman D. Recovery colleges in mental health-care services: an Australian feasibility and acceptability study. *Mental Health and Social Inclusion* 2022; **26**(1): 12-22.	Australia
15	Kay K, Edgley G. Evaluation of a new recovery college: delivering health outcomes and cost efficiencies via an educational approach. *Mental Health and Social Inclusion* 2019; **23**(1): 36-46.	UK
16	Khan BM, Reid N, Brown R, Kozloff N, Stergiopoulos V. Engaging Adults Experiencing Homelessness in Recovery Education: A Qualitative Analysis of Individual and Program Level Enabling Factors. *Frontiers in Psychiatry* 2020; **11**.	Canada
*17	King T, Meddings S. Survey identifying commonality across international Recovery Colleges. *Mental Health and Social Inclusion* 2019; **23**(3): 121-8.	International
18	Lucchi F, Chiaf E, Placentino A, Scarsato G. Programma FOR: A Recovery College in Italy. *Journal of Recovery in Mental Health* 2018; **1**(3): 29-37.	Italy
19	Meddings S, Guglietti S, Lambe H, Byrne D. Student perspectives: recovery college experience. *Mental Health and Social Inclusion* 2014; **18**(3): 142-50.	UK

20	Meddings S, Campbell E, Guglietti S, et al. From service user to student: The benefits of recovery college. *Clinical Psychology Forum* 2015; (268): 32–7.	UK
21	Meddings S, Walsh L, Patmore L, McKenzie KLE, Holmes S. To what extent does Sussex Recovery College reflect its community? An equalities and diversity audit. *Mental Health and Social Inclusion* 2019; **23**(3): 136-44.	UK
22	Muir-Cochrane E, Lawn S, Coveney J, Zabeen S, Kortman B, Oster C. Recovery college as a transition space in the journey towards recovery: An Australian qualitative study. *Nursing & Health Sciences* 2019; **21**(4): 523-30.	Australia
23	Newman-Taylor K, Stone N, Valentine P, Hooks Z, Sault K. The Recovery College: A unique service approach and qualitative evaluation. *Psychiatr Rehabil J* 2016; **39**(2): 187-90.	UK
24	Nurser K, Hunt D, Bartlett T. Do recovery college courses help to improve recovery outcomes and reduce self-stigma for individuals who attend? *Clinical Psychology Forum* 2017; **300**: 32–7.	UK
25	Peer JE, Gardner M, Autrey S, Calmes C, Goldberg RW. Feasibility of implementing a recovery education center in a Veterans Affairs medical center. *Psychiatr Rehabil J* 2018; **41**(2): 135-40.	United States
26	Reid N, Castel S, Veldhuizen S, Roberts A, Stergiopoulos V. Effect of a Psychiatric Emergency Department Expansion on Acute Mental Health and Addiction Service Use Trends in a Large Urban Center. *Psychiatric Services* 2019; **70**(11): 1053-6.	Canada
27	Sommer J, Gill K, Stein-Parbury J. Walking side-by-side: Recovery Colleges revolutionising mental health care. *Mental Health and Social Inclusion* 2018; **22**(1): 18-26.	Australia
28	Sommer J, Gill KH, Stein-Parbury J, Cronin P, Katsifis V. The role of recovery colleges in supporting personal goal achievement. *Psychiatric Rehabilitation Journal* 2019; **42**(4): 394.	Australia
29	Stevens J, Butterfield C, Whittington A, Holttum S. Evaluation of Arts based Courses within a UK Recovery College for People with Mental Health Challenges. *International Journal of Environmental Research and Public Health*, 2018; **15**(6): 1170.	UK
30	Sutton R, Lawrence K, Zabel E, French P. Recovery College influences upon service users: a Recovery Academy exploration of employment and service use. *Journal of Mental Health Training, Education & Practice* 2019; **14**(3): 141-8.	UK
31	Thompson H, Simonds L, Barr S, Meddings S. Recovery colleges: long-term impact and mechanisms of change. *Mental Health and Social Inclusion* 2021; **25**(3): 232-42.	UK
32	Wilson C, King M, Russell J. A mixed-methods evaluation of a Recovery College in South East Essex for people with mental health difficulties. *Health & Social Care in the Community* 2019; **27**(5): 1353-62.	Canada
33	Windsor L, Roberts G, Dieppe P. Recovery Colleges – safe, stimulating and empowering. *Mental Health and Social Inclusion* 2017; **21**(5): 280-8.	UK

34	Zabel E, Donegan G, Lawrence K, French P. Exploring the impact of the recovery academy: a qualitative study of Recovery College experiences. *The Journal of Mental Health Training, Education and Practice* 2016; **11**(3): 162-71.	UK
35	Secker J, Wilson C. Evaluation of the mid Essex recovery college, October–December 2013. Chelmsford: Anglia Ruskin University, 2014.	UK
36	Arbour S, Stevens A, Gasparini J. Recovery College: Influencing recovery-related outcomes. Culture and System Transformation [PowerPoint] 2019.	Canada
37	Barton S, Williams R. Evaluation of “the exchange” (Barnsley Recovery College). https://rfact.org.au/wp-content/uploads/ 2015/05/Evaluation-of-The-Exchange-Barnsley-Recovery-College- SWYFT.pdf 2015.	UK
38	Canadian Mental Health Association. Recovery College: *White paper*. Canadian Mental Health Association 2020.	Canada
39	Kaminskiy E. Moore S. *South Essex Recovery College evaluation project report* (pp. 1–53). https://arro.anglia.ac.uk/id/eprint/ 600456 2015.	UK
40	Kenny B. Kavanagh E. McSherry H. Brady G. Kelly J. MacGabhann L. Griffin M. Farrelly M. Kelly N. Ross P. Barron R. Watters R. Keating S. *Informing and transforming communities, shaping the way forward for mental health recovery*. Dublin North. North East Recovery College 2020.	Ireland
41	Mayo Recovery College. *My words, my way*. Mayo Recovery College n.d.	Ireland
42	Taylor D. Boland A. Wallace N. *Mid West ARIES project: A report on the development, progress and outcomes of a pilot project to provide a recovery education service in the Mid West*. University of Limerick 2017.	Ireland
43	Wels J. *Recovery College Greenwich – Evaluation report*. https://www.bridgesupport.org/wp-content/uploads/2019/01/Enc-4.1- Greenwich-University-Evaluation-Report-August-2016-Final.pdf 2016.	UK

*Same study as No. 6 in Theriault’s review in 2020 (full reference below), which categorised the location of this study being in England.

Thériault J, Lord M-M, Briand C, Piat M, Meddings S. Recovery Colleges After a Decade of Research: A Literature Review. *Psychiatric Services* 2020; **71**(9): 928-40.

Included sources in Thériault J, Lord M-M, Briand C, Piat M, Meddings S. Recovery Colleges After a Decade of Research: A Literature Review. *Psychiatric Services* 2020; **71**(9): 928-40.

	Authors	**Country**
1	Anfossi A. *The current state of Recovery Colleges in the UK*. Nottingham, UK, Implementing Recovery Through Organisational Change 2017	England
2	Bourne P, Meddings S, Whittington A. An evaluation of service use outcomes in a Recovery College. *Journal of Mental Health* 2018; **27**(4): 359-66.	England
3	Chung TE, Eckerle Curwood S, Thang H, Gruszecki S, Beder M, Stergiopoulos V. Introducing a Recovery Education Centre for Adults Experiencing Mental Health Challenges and Housing Instability in a Large Urban Setting. *International Journal of Mental Health and Addiction* 2016; **14**(5): 850-5.	Canada
4	Dunn EC, Sally Rogers E, Hutchinson DS, et al. Results of an Innovative University-based Recovery Education Program for Adults with Psychiatric	England

	Disabilities. *Administration and Policy in Mental Health and Mental Health Services Research* 2008; **35**(5): 357-69.	
5	Kay K, Edgley G. Evaluation of a new recovery college: delivering health outcomes and cost efficiencies via an educational approach. *Mental Health and Social Inclusion* 2019; **23**(1): 36-46.	England
*6	King T, Meddings S. Survey identifying commonality across international Recovery Colleges. *Mental Health and Social Inclusion* 2019; **23**(3): 121-8.	England
7	Meddings S, Campbell E, Guglietti S, et al. From service user to student: The benefits of recovery college. *Clinical Psychology Forum* 2015; (268): 32–7.	England
8	Meddings S, Walsh L, Patmore L, McKenzie KLE, Holmes S. To what extent does Sussex Recovery College reflect its community? An equalities and diversity audit. *Mental Health and Social Inclusion* 2019; **23**(3): 136-44.	England
9	Nurser K, Hunt D, Bartlett T. Do recovery college courses help to improve recovery outcomes and reduce self-stigma for individuals who attend? *Clinical Psychology Forum* 2017; **300**: 32–7.	England
10	Sommer J, Gill KH, Stein-Parbury J, Cronin P, Katsifis V. The role of recovery colleges in supporting personal goal achievement. *Psychiatric Rehabilitation Journal* 2019; **42**(4): 394.	Australia
11	Burhouse A, Rowland M, Marie Niman H, et al. Coaching for recovery: a quality improvement project in mental healthcare. *BMJ Quality Improvement Reports* 2015; **4**(1): u206576.w2641.	England
12	Ebrahim S, Glascott A, Mayer H, Gair E. Recovery Colleges; how effective are they? *The Journal of Mental Health Training, Education and Practice* 2018; **13**(4): 209-18.	England
13	Hall T, Jordan HL, Reifels L, et al. A Process and Intermediate Outcomes Evaluation of an Australian Recovery College. *Journal of Recovery in Mental Health* 2018; **1**(3): 7-20.	Australia
14	Hopkins L, Pedwell G, Lee S. Educational outcomes of Discovery College participation for young people. *Mental Health and Social Inclusion* 2018; **22**(4): 195-202.	Australia
15	Lucchi F, Chiaf E, Placentino A, Scarsato G. Programma FOR: A Recovery College in Italy. *Journal of Recovery in Mental Health* 2018; **1**(3): 29-37.	Italy
16	Meddings S, Guglietti S, Lambe H, Byrne D. Student perspectives: recovery college experience. *Mental Health and Social Inclusion* 2014; **18**(3): 142-50.	England
17	Perkins AM, Ridler JH, Hammond L, Davies S, Hackmann C. Impacts of attending recovery colleges on NHS staff. *Mental Health and Social Inclusion* 2017; **21**(1): 18-24.	England
18	Stevens J, Butterfield C, Whittington A, Holttum S. Evaluation of Arts based Courses within a UK Recovery College for People with Mental Health Challenges. *International Journal of Environmental Research and Public Health*, 2018; **15**(6): 1170.	England
19	Cameron J, Hart A, Brooker S, Neale P, Reardon M. Collaboration in the design and delivery of a mental health Recovery College course: experiences of students and tutors. *J Ment Health* 2018; **27**(4): 374-81.	England
20	Crowther A, Taylor A, Toney R, et al. The impact of Recovery Colleges on mental health staff, services and society. *Epidemiology and Psychiatric Sciences* 2019; **28**(5): 481-8.	England
21	Dalgarno M, Oates J. The meaning of co-production for clinicians: An exploratory case study of Practitioner Trainers in one Recovery College. *Journal of Psychiatric and Mental Health Nursing* 2018; **25**(5-6): 349-57.	England
22	Frayn E, Duke J, Smith H, Wayne P, Roberts G. A voyage of discovery: setting up a recovery college in a secure setting. *Mental Health and Social Inclusion* 2016; **20**(1): 29-35.	England

23	Gill KH. Recovery colleges, co-production in action: The value of the lived experience in “learning and growth for mental health”. *Health Issues* 2014; (113): 10-4.	Australia
24	Harper L, McKeown M. Why make the effort? Exploring recovery college engagement. *Mental Health and Social Inclusion* 2018; **22**(1): 27-33.	England
25	Martina T. Poetry for recovery: Peer trainer reflections at Sussex Recovery College. *Clinical psychology forum* 2015; **268**: 28-31.	England
26	Newman-Taylor K, Stone N, Valentine P, Hooks Z, Sault K. The Recovery College: A unique service approach and qualitative evaluation. *Psychiatr Rehabil J* 2016; **39**(2): 187-90.	England
27	Nurser K, Rushworth I, Shakespeare T, Williams D. Personal storytelling in mental health recovery. *Mental Health Review Journal* 2018; **23**(1): 25-36.	England
28	Sommer J, Gill K, Stein-Parbury J. Walking side-by-side: Recovery Colleges revolutionising mental health care. *Mental Health and Social Inclusion* 2018; **22**(1): 18-26.	Australia
29	Toney R, Elton D, Munday E, et al. Mechanisms of Action and Outcomes for Students in Recovery Colleges. *Psychiatric Services* 2018; **69**(12): 1222-9.	England
30	Windsor L, Roberts G, Dieppe P. Recovery Colleges – safe, stimulating and empowering. *Mental Health and Social Inclusion* 2017; **21**(5): 280-8.	England
31	Zabel E, Donegan G, Lawrence K, French P. Exploring the impact of the recovery academy: a qualitative study of Recovery College experiences. *The Journal of Mental Health Training, Education and Practice* 2016; **11**(3): 162-71.	England

*Same study as No. 17 in Lin’s review in 2022 (full reference below), which categorised this study as an international study.

Lin E, Harris H, Black G, et al. Evaluating recovery colleges: a co-created scoping review. *Journal of Mental Health* 2022: 1-22.

Included sources in
Crowther A, Taylor A, Toney R, et al. The impact of Recovery Colleges on mental health staff, services and society. *Epidemiology and Psychiatric Sciences* 2019; **28**(5): 481-8., and
Toney R, Elton D, Munday E, et al. Mechanisms of Action and Outcomes for Students in Recovery Colleges. *Psychiatric Services* 2018; **69**(12): 1222-9. (Same 44 sources)

	**Authors**	**Country**
1	Frayn E, Duke J, Smith H, Wayne P, Roberts G. A voyage of discovery: setting up a recovery college in a secure setting. *Mental Health and Social Inclusion* 2016; **20**(1): 29-35.	UK
2	P erkins R, Repper J. When is a “recovery college” not a “recovery college”? *Mental Health and Social Inclusion* 2017; **21**(2): 65-72.	UK
3	Meddings S, Campbell E, Guglietti S, et al. From service user to student: The benefits of recovery college. *Clinical Psychology Forum* 2015; (268): 32–7.	UK
4	Taggart H, Kempton J. The Route to Employment: The Role ofMental Health Recovery Colleges. London: CentreForum; 2015.	UK
5	North Essex Research Network. Evaluation of the Mid Essex Recovery College October–December 2013. Essex: North Essex Research Network; 2014.	UK
6	The Dorset Wellbeing and Recovery Partnership (WaRP). WaRP Magazine. 2016.	UK
7	McGregor J, Repper J, Brown H. “The college is so different from anything I have done”. A study of the characteristics of Nottingham Recovery College. *The Journal of Mental Health Training, Education and Practice* 2014; **9**(1): 3-15.	UK
8	Oh H. The pedagogy of recovery colleges: clarifying theory. *Mental Health Review Journal* 2013; **18**(4).	UK

9	Skipper L, Page K. Our recovery journey: two stories of change within Norfolk and Suffolk NHS Foundation Trust. *Mental Health and Social Inclusion* 2015; **19**(1): 38-44.	UK
10	Watson E. What Makes a Recovery College? A Systematic LiteratureReview of Recovery Education in Mental Health [Dissertation]. Nottingham: University of Nottingham; 2013.	UK
11	Kelly J, Gallagher S, McMahon J. Developing a recovery college: a preliminary exercise in establishing regional readiness and community needs. *Journal of Mental Health* 2017; **26**(2): 150-5.	UK
12	Dunn EC, Sally Rogers E, Hutchinson DS, et al. Results of an Innovative University-based Recovery Education Program for Adults with Psychiatric Disabilities. *Administration and Policy in Mental Health and Mental Health Services Research* 2008; **35**(5): 357-69.	UK
13	Hall T, Brophy L, Jordan H. A report on the early outcomes of the Mind Recovery College. Melbourne: Centre for Mental Health, Melbourne School of Population and Global Health, 2016.	Australia
14	Hall T, Brophy L, Jordan H, et al. Co-Producing The Journey To Recovery: The Mind Recovery College, Australia. TheMHS Conference 2016; 2016; Auckland, Australia: TheMHS Learning Network Inc.; 2016.	Australia
15	McGregor J, Brophy L, Hardy D, et al. Proceedings of June 2015 Meeting. Recovery Colleges International Community of Practice (RCICoP); 2015; 2015.	UK
16	Mind. Australasian Recovery College Community of Practice Inaugural Meeting. Victoria: Mind; 2016.	Australia
17	Newman-Taylor K, Stone N, Valentine P, Hooks Z, Sault K. The Recovery College: A unique service approach and qualitative evaluation. *Psychiatr Rehabil J* 2016; **39**(2): 187-90.	UK
18	Shepherd G, McGregor J. Recovery Colleges – Evolution or Revolution?. Ghent, November 9; 2016.	UK
19	Sussex Recovery College. Performance and Evaluation Report (Summer Term 2016); Brighton: Sussex Partnership NHS Foundation Trust; 2016	UK
20	Thornhill H, Dutta A. Are recovery colleges socially acceptable? *BJPsych International* 2016; **13**(1): 6-7.	UK
21	Zabel E, Donegan G, Lawrence K, French P. Exploring the impact of the recovery academy: a qualitative study of Recovery College experiences. *The Journal of Mental Health Training, Education and Practice* 2016; **11**(3): 162-71.	UK
22	Burhouse A, Rowland M, Marie Niman H, et al. Coaching for recovery: a quality improvement project in mental healthcare. *BMJ Quality Improvement Reports* 2015; **4**(1): u206576.w2641.	UK
23	Central and North West London NHS Foundation Trust (2015) CNWL Recovery & Wellbeing College Annual Report April 2014 - July 2015.	UK
24	Kaminskiy E, Moore S. South essex recovery college evaluation. Chelmsford, UK.: Anglia Ruskin University, 2015.	UK
25	King T. An exploratory study of co-production in recovery colleges in the UK. Sussex: University of Brighton.; 2015.	UK
26	Meddings S, Campbell E, Guglietti S, et al. From service user to student: The benefits of recovery college. *Clinical Psychology Forum* 2015; (268): 32–7.	UK
27	Gill KH. Recovery colleges, co-production in action: The value of the lived experience in "learning and growth for mental health". *Health Issues* 2014; (113): 10-4.	Australia
28	McCaig M, McNay L, Marland G, Bradstreet S, Campbell J. Establishing a recovery college in a Scottish University. *Mental Health and Social Inclusion* 2014; **18**(2): 92-7.	UK

29	McMahon J, Wallace N, Kelly J, Egan E. Recovery education college: a needs analysis. Dublin: Health Service Executive, 2014.	Ireland
30	Meddings S, Byrne D, Barnicoat S, Campbell E, Locks L. Co-delivered and co-produced: creating a recovery college in partnership. *The Journal of Mental Health Training, Education and Practice* 2014; **9**(1): 16-25.	UK
31	Meddings S, Guglietti S, Lambe H, Byrne D. Student perspectives: recovery college experience. *Mental Health and Social Inclusion* 2014; **18**(3): 142-50.	UK
32	Rennison J, Skinner S, Bailey A. CNWL Recovery College Annual Report April 2013 - March 2014. London: Central and North West London NHS Foundation Trust., 2014.	UK
33	Alois Zucchelli F, Skinner S. Central and North West London NHS Foundation Trust's (CNWL) Recovery College: the story so far …. *Mental Health and Social Inclusion* 2013; **17**(4): 183-9.	UK
34	Mind. Establishment of the Mind Recovery College. Heidelberg: Mind Australia, 2012.	Australia
35	Perkins R, Repper J, Rinaldi M, Brown H. Recovery Colleges. London: Implementing Recovery Through Organisational Change, 2012.	UK
36	Rinaldi M, Morland M, Wybourn S. Annual Report 2011 – 2012 South West London Recovery College, London, South West London and St George's Mental Health NHS Trust. London: National Health Service, 2012.	UK
37	Rinaldi M, Suleman M. Care co-ordinators' attitudes to self-management and their experience of the use of the South West London Recovery College. London: South West London and St George's Mental Health NHS Trust.; 2012	UK
38	Rinaldi M, Wybourn S. The Recovery College Pilot in Merton and Sutton: longer term individual and service level outcomes. London: South West London and St. Georges Mental Health NHS Trust.; 2011.	UK
39	Bourne P, Meddings S, Cooper R, Locks L, Whittington A. An evaluation of service use outcomes in Sussex Recovery College. . Sussex: Sussex NHS Trust; 2016.	UK
40	Bristow E. An annual report of Lincoln Recovery College. Lincoln: Lincolnshire Partnership NHS Foundation Trust, 2015.	UK
41	Martina T. Poetry for recovery: Peer trainer reflections at Sussex Recovery College. *Clinical psychology forum* 2015; **268**: 28-31.	UK
42	Solent Recovery College. Solent Recovery College, Our first year – Outcomes. . Portsmouth: Solent Recovery College; 2014.	UK
43	Barton, South West Yorkshire Partnership NHS Foundation Trust Recovery College [PowerPoint] n.d.	UK
44	Sault, Garner, Gatherer, Southern Health Recovery College [PowerPoint] n.d.	UK

Included sources in Toney R, Knight J, Hamill K, et al. Development and Evaluation of a Recovery College Fidelity Measure. *The Canadian Journal of Psychiatry* 2018; **64**(6): 405-14

No	Authors	Country
1	McGregor J, Repper J, Brown H. "The college is so different from anything I have done". A study of the characteristics of Nottingham Recovery College. *The Journal of Mental Health Training, Education and Practice* 2014; **9**(1): 3-15.	UK
2	Perkins R, Repper J, Rinaldi M, Brown H. Briefing: Recovery Colleges2012. https://imroc.org/wp-content/uploads/2022/04/1.Recovery-Colleges.pdf (accessed 23 January 2023).	UK
3	Mind. Australasian Recovery College Community of Practice Inaugural Meeting. Victoria: Mind; 2016.	Australia
4	Meddings S, Campbell E, Guglietti S, et al. From service user to student: The benefits of recovery college. *Clinical Psychology Forum* 2015; (268): 32–7.	UK

5	Shepherd G, McGregor J, Meddings S, Roeg W. Recovery colleges and co-production. *Wellbeing, recovery and mental health* 2017: 181-93.	UK
6	Perkins AM, Ridler JH, Hammond L, Davies S, Hackmann C. Impacts of attending recovery colleges on NHS staff. *Mental Health and Social Inclusion* 2017; **21**(1): 18-24.	UK
7	Shepherd G, Boardman J, Rinaldi M, Roberts G. Supporting recovery in mental health services: Quality and outcomes. *London: Implementing Recovery Through Organisational Change* 2014.	UK
8	Leamy M, Bird V, Boutillier CL, Williams J, Slade M. Conceptual framework for personal recovery in mental health: systematic review and narrative synthesis. *British Journal of Psychiatry* 2011; **199**(6): 445-52.	UK
9	McGregor J, Brophy L, Hardy D, et al. Proceedings of June 2015 Meeting. Recovery Colleges International Community of Practice (RCICoP); 2015; 2015.	UK
10	Rinaldi M, Morland M, Wybourn S. Annual Report 2011 – 2012 South West London Recovery College, London, South West London and St George's Mental Health NHS Trust. London: National Health Service, 2012.	UK
11	Watson E. What Makes a Recovery College? A Systematic LiteratureReview of Recovery Education in Mental Health [Dissertation]. Nottingham: University of Nottingham; 2013.	UK
12	Meddings S, Guglietti S, Lambe H, Byrne D. Student perspectives: recovery college experience. *Mental Health and Social Inclusion* 2014; **18**(3): 142-50.	UK
13	King T. An exploratory study of co-production in recovery colleges in the UK. Sussex: University of Brighton.; 2015.	UK

Supplementary Information 2. Inclusion criteria for Recovery Colleges

Currently active services that:	
(a)	Targeting to supporting personal recovery, defined as living a purposeful and autonomous life despite the presence of mental health symptom
(b)	Prioritising co-production, defined as individuals with lived experience working with staff or subject experts to design and deliver all aspects of the Recovery College
(c)	Prioritising adult learning approaches, in which students and trainers work together and learn from each other by sharing experiences, knowledge, and skills

These criteria were confirmed by the service managers, who were approached by the research team in the England survey, and by each country lead in the international survey.
